# 

**Published:** 1923-06

**Authors:** 


### Contents

Event and Comment .......... 241
Expect 10,000 Dentists to Attend Convention .......... 241
Seventy-Seventh Annual Commencement .......... 261
Baccalaureate Address .......... 264
Tuberculosis of the Intestine .......... 267
American Dental Association .......... 277
Cleaning Pennsylvania's Mouth .......... 278
Announcements .......... 283
Book Reviews .......... 284
Indents .......... 286

